# A Rare Case of Pheohyphomycotic Lumbar Spondylodiscitis Mistreated as Koch's Spine

**DOI:** 10.1155/2016/8705204

**Published:** 2016-12-18

**Authors:** Shakti A. Goel, Hitesh N. Modi, Yatin J. Desai, Harshal P. Thaker

**Affiliations:** ^1^Department of Orthopaedics and Spine Surgery, Zydus Hospitals and Healthcare Research Pvt. Ltd., Thaltej, Ahmedabad, Gujarat, India; ^2^Dr. Harshal Thaker's Clinic, Ambawadi, Ahmedabad, Gujarat, India

## Abstract

Pheohyphomycosis is an uncommon infection and its association in spondylodiscitis has not yet been reported. The purpose of this case report is to describe a rare case of Pheohyphomycotic spondylodiscitis and methods to diagnose and manage the patient with less invasive techniques. A 29-year-old male patient presented to the outpatient department with complaints of gradually increasing low back pain with bilateral lower limbs radicular pain since one and a half years. He had associated fever, weight loss, voice changes, and dry, scaly, erythematous skin with elevated ESR. The patient had been taking anti-Koch's therapy since 1 year with little relief in pain and no radiological improvement. Percutaneous pedicle biopsy of L4 vertebra was taken under local anaesthesia and confirmed Pheohyphomycosis which was treated with antifungal medications. The patient showed sequential improvement with long term antifungal treatment. He was eventually able to walk independently without support.

## 1. Introduction

Pheohyphomycosis represents infections caused by pigmented filamentous fungi which contain melanin in their cell walls [1]. Their morphologic characteristics include hyphae, yeast-like cells, or combination [2]. They are either associated with* Alternaria or Exophiala jeanselmei* [3, 4].

Pheohyphomycosis is an uncommon infection and almost all reported cases have occurred in immunosuppressed patients with 80 percent mortality rate [5]. The disease is transmissible through air, wind, and water. The contacted individuals and population can be easily affected by it and usually it is too late to be successfully treated by the time the disease is recognized. Whenever seen, these organisms affect the skin and subcutaneous tissue with nodules or cyst [6]. Eye infections and plaques and granulomatous damage on the body have also been reported [7]. However, effect on spine by this organism in Southern Asia has not yet been seen.

Here we report a unique case of Pheohyphomycosis of lumbosacral spine, previously mistaken for Koch's spine (TB) and treated accordingly. The patient did not report any improvement for a long time until the pedicle biopsy was taken and antifungal treatment started.

Tuberculosis (TB) has been described as an ancient infectious disease with evidence being discovered in centuries-old skeletal remains [8]. Moreover, it is the most common cause of spinal infection in south Asian population [9]. In recent decades, there has been a significant resurgence of TB (Tuberculosis), causing 2-3 million deaths annually worldwide [10, 11].

Due to the frequent prevalence of TB in spine, it is a common practice to consider Koch's spine (TB) as the first differential in all spinal infections in developing nations. Many of these patients get better with anti-Koch's therapy without the surgery [12]. Hence it is a common practice to start anti-Koch's therapy in patients while the biopsy report is awaited [12]. However, rarely, other organisms besides TB could be a reason for such an infection [13]. This is the first case report of Pheohyphomycosis causing spinal infection in an immunocompetent host.

## 2. Case Report

A 29-year-old immunocompetent male patient presented to the outpatient department with complaints of gradually increasing low back pain since one and a half years. The pain increased on changing position and relieved with rest. There was associated bilateral lower limb radiculopathy which was more on right side as compared to left. Manual muscle testing of upper and lower limbs did not show any reduction in the grade of power but right lower limb straight leg raising was restricted to 50 degrees. Radical involvement of L4, L5, and S1 nerve root was seen. However, there was no motor weakness present. The patient had radicular pain to right lower limb in posterior gluteal region, lateral and anterior shin. The sensations were intact in all four limbs and reflexes were of two plus grade with a flexor Babinski and negative Hoffman's test. He had associated fever and weight loss of seven kilograms in one year. There were associated voice changes and dry, scaly, erythematous skin, the biopsy of which was previously reported to be inconclusive of any infection. He had lumbar kyphosis and elevated ESR (Erythrocyte Sedimentation Rate) of 94 mm per hour. The patient had been taking anti-Koch's therapy since 1 year with little relief in pain.

**Figure 1:**
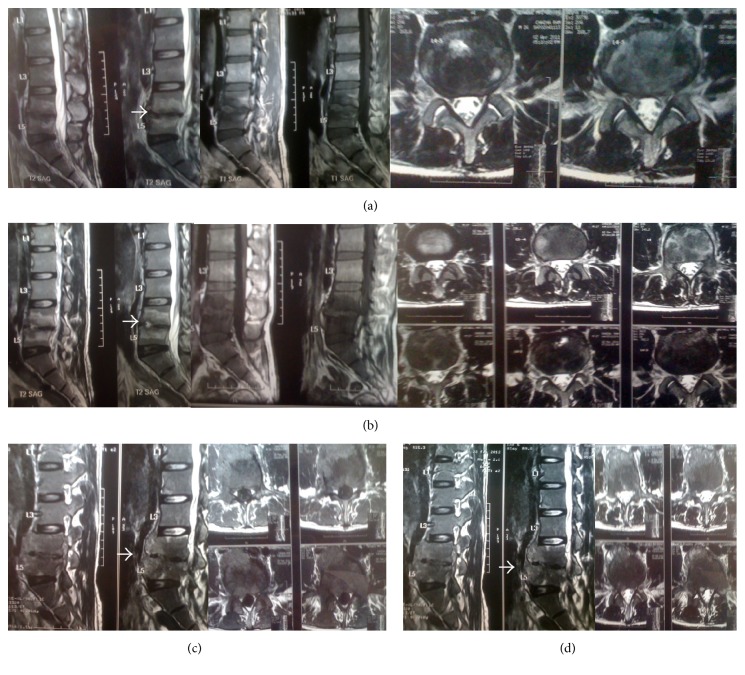
Sequential MRI images of the patient. (a) MRI before the initiation of anti-Koch's therapy. (b) MRI image after 4 months of anti-Koch's therapy. (c) MRI image after 9 months of anti-Koch's therapy. (d) MRI image after 12 months of anti-Koch's therapy.

The patient had a series of MRI (Magnetic Resonance Imaging) images starting with the first one taken 14 months ago (Figure 1(a)). It showed spondylodiscitis at lumbar 4 and lumbar 5 region and patient was started on anti-Koch's therapy. The second MRI taken after 4 months of anti-Koch's therapy did not show any improvement in the disc lesion or symptoms. The lesion rather progressed in the second MRI (Figure 1(b)). The third MRI taken 9 months after anti-Koch's therapy treatment did not even show any improvement (Figure 1(c)). The fourth MRI was taken after the patient completed 1 year of anti-Koch's therapy (Figure 1(d)). It showed further destruction of lumbar 4-lumbar 5 disc areas and bodies. The lesion had further progressed and patient reported no improvement in symptoms.

**Figure 2:**
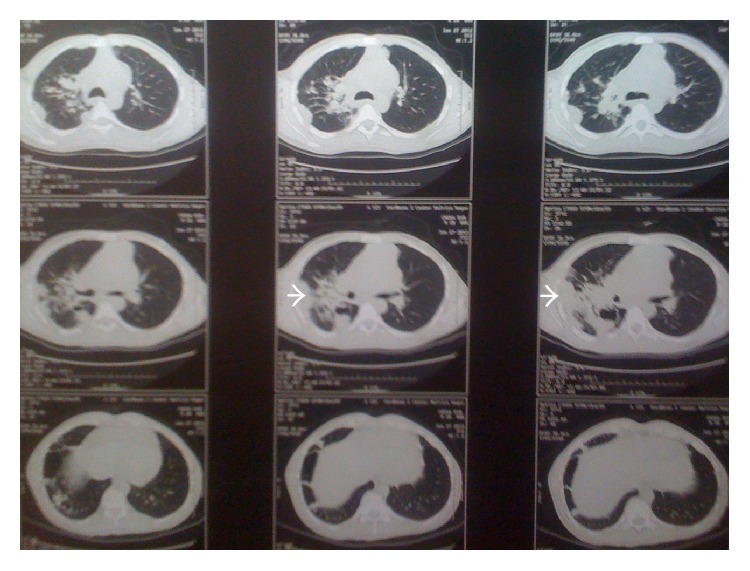
CT scan of the chest showing lesion in the lungs. Biopsy of these lesions was inconclusive.

Considering other differentials like plasmacytoma, lymphocytoma, fungal infection, or metastasis in mind, CT (Computerised Tomography) scan was advised. It showed lesions in the lung and liver (Figure 2). CT guided biopsy of the lung was done which was inconclusive. No specific pathology in the CT scan of the abdomen and pelvis was found.

**Figure 3:**
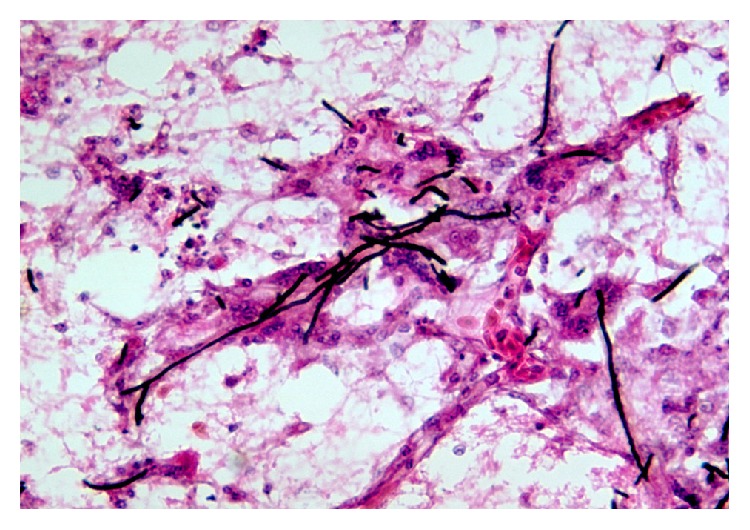
Microscopic image (Silver Methenamine, Haemotoxylin and Eosin Staining) of the 4th pedicle biopsy showing hyphae and yeast-like cells suggesting Pheohyphomycosis. The biopsy material was stained with Silver Methenamine with H&E counterstaining. Gram-positive hyphae/pseudohyphae can be appreciated.

**Figure 4:**
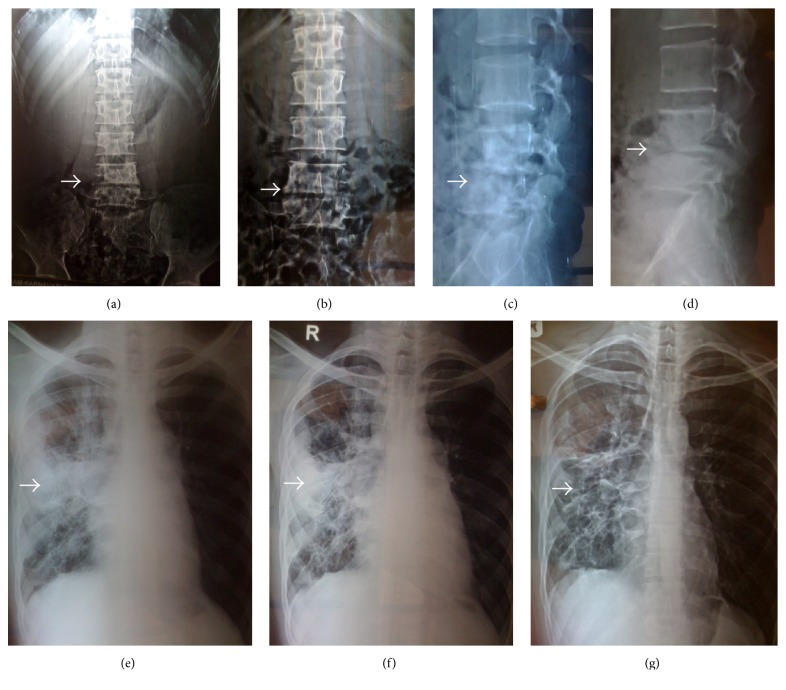
Radiographic evidence of improvement in spine and chest after antifungal treatment. (a) and (b) represent anteroposterior X-rays of lumbar spine, before and after treatment, respectively. (c) and (d) represent lateral X-rays of lumbar spine, before and after treatment, respectively. (e), (f), and (g) represent the sequential improvement in chest radiographs after the initiation of antifungal treatment.

Following CT scan, it was decided to take 4th lumbar vertebra's pedicle biopsy under local anaesthesia and look for culture sensitivity and fungal growth. The microscopy showed hyphae and yeast-like cells representing Pheohyphomycosis (Figure 3). The patient was started on intravenous antifungal medications for 2 weeks (Amphotericin B 3–5 mg/Kg body weight). This was followed by 12 months of oral antifungal medications (tablet Voriconazole twice a day). The leg pain of the patient disappeared in one week. The back pain relieved by 90 percent in one month and he started to walk independently without support. The improvements in the radiographs of chest and spine were also evident (Figure 4). At present the patient has no mechanical pain symptoms and is walking independently without support.

## 3. Discussion

Tuberculosis has been reported to be the commonest cause of spondylodiscitis. Other causes of spinal infection have also been reported [13]. This is the first case report which shows Pheohyphomycosis as a cause of spondylodiscitis in an immunocompetent host which was diagnosed with percutaneous pedicle biopsy.

Pheohyphomycosis is an amalgam of clinical diseases caused by a variety of fungi which often lead to subcutaneous cyst formation at the site of traumatic implantation [1, 3]. It is more commonly found in immunocompromised host; however skin lesion in immunocompetent adults has also been reported. Primary pulmonary infection is usually associated with an endemic area and with the inhalation of a large number of infecting spores. Normal patients exposed to such fungi generally fight off infection easily with no sign of illness. However, if exposed to a large enough inoculum of virulent fungus, a person with an intact immune system may develop a chronic infection. The chronic infection may require treatment such as antifungal drugs and surgery and require the patient to abstain from smoking. A compromised patient may easily develop systemic and progressive illness depending on the specifics surrounding the infection [6, 7]. In this case, the patient was an immunocompetent adult, in contrast to the usual findings of Pheohyphomycotic fungal infection. Moreover, though he did not have any respiratory complaints, the lungs did confirm the fungal lesion on CT scan. This further lays emphasis on the usual findings of the fungal spore entry via pulmonary route.

There is little information on spinal Pheohyphomycosis management. However, based on available data and experience, therapy with Amphotericin B alone may not be adequate. Some successfully treated cases of cerebral Pheohyphomycosis used itraconazole or flucytosine in combination with Amphotericin B, although few have been documented. The newer azole antifungal drugs such as Voriconazole and posaconazole may also play a role in therapy [14, 15]. Prolonged follow-up is essential because relapses are not uncommon [15]. In this case study, the patient was started on intravenous antifungal medications for 2 weeks (Amphotericin B 3–5 mg/Kg body weight). This was followed by 12 months of oral antifungal medications (tablet Voriconazole twice a day). This long term therapy made the patient symptom-free and he started to walk independently without support.

Two important questions have been raised by this case study. First one is if biopsy confirmation is a must after radiological signs of infection in spine before starting the treatment. The pedicle biopsy can be taken by an open technique under general anaesthesia or percutaneous method under local anaesthesia. The percutaneous pedicle biopsy under local anaesthesia is a promising alternative as described fby Dave et al. [16]. In this case study, the biopsy taken was by percutaneous method under local anaesthesia. The importance of this technique lies in the fact that there is a definitive evidence of the microorganism and the patient is not mistreated with inappropriate antimicrobials. The patient in this case study was mistreated as Koch's spine until pedicle biopsy confirmed Pheohyphomycosis as the cause of the disease.

The second question that arises is if anti-Koch's therapy should be started in all patients with spinal infections in Asian nations. It is a common practice to start anti-Koch's therapy in all spondylodiscitis patients and wait for the response in Southern Asia while results of biopsy are awaited or negative [12]. A delay in diagnosing such an infection and mistreating it as Koch's spine will not only keep the patient in pain for a long time but also make him suffer the toxic side effects of anti-Koch's therapy [17, 18].

Here we report a unique case of Pheohyphomycotic spondylodiscitis which was diagnosed and treated by a nonsurgical method. The pedicle biopsy of the 4th lumbar vertebra was taken under local anaesthesia [16]. The patient showed complete recovery after the diagnosis and medical treatment of the fungal infection.

## 4. Conclusion

A number of differentials can be kept in mind while encountering a case of spondylodiscitis. Besides tuberculosis, plasmacytoma, lymphocytoma, metastasis, chronic nonspecific inflammations, and fungal infections should be considered. It is at the physician's discretion to decide about the next modality of treatment. Should the patient be started on anti-Koch's therapy in south Asian region as it is routinely done or should results of open biopsy/surgery/radiochemotherapy be awaited? Percutaneous pedicle biopsy under local anaesthesia is an efficient alternative.

Here we present a unique case of Pheohyphomycotic spondylodiscitis of lumbar 4-lumbar 5 region which was misdiagnosed and treated as Koch's spine until percutaneous lumbar 4 pedicle biopsy confirmed the diagnosis and patient was treated with antifungal medications.

## References

[1] Ajello L., Georg L. K., Steigbigel R. T., Wang C. J. (1974). A case of phaeohyphomycosis caused by a new species of Phialophora. *Mycologia*.

[2] James W. D. (2006). Rosacea: wonderings of a clinician. *Cutis*.

[3] Boyce R., Deziel P., Otley C. (2010). Phaeohyphomycosis due to Alternaria species in transplant recipients. *Transplant Infectious Disease*.

[4] Umemoto N., Demitsu T., Kakurai M. (2009). Two cases of cutaneous phaeohyphomycosis due to exophiala jeanselmei: diagnostic significance of direct microscopical examination of the purulent discharge. *Clinical and Experimental Dermatology*.

[5] Revankar S. G., Patterson J. E., Sutton D. A., Pullen R., Rinaldi M. G. (2002). Disseminated phaeohyphomycosis: review of an emerging mycosis. *Clinical Infectious Diseases*.

[6] Hasei M., Takeda K., Anzawa K., Nishibu A., Tanabe H., Mochizuki T. (2013). Case of phaeohyphomycosis producing sporotrichoid lesions. *Journal of Dermatology*.

[7] Cai Q., Lv G.-X., Jiang Y.-Q. (2013). The first case of phaeohyphomycosis caused by *Rhinocladiella basitona* in an immunocompetent child in China. *Mycopathologia*.

[8] Kiran N. A. S., Vaishya S., Kale S. S., Sharma B. S., Mahapatra A. K. (2007). Surgical results in patients with tuberculosis of the spine and severe lower-extremity motor deficits: a retrospective study of 48 patients. *Journal of Neurosurgery: Spine*.

[9] Rasouli M. R., Mirkoohi M., Vaccaro A. R., Yarandi K. K., Rahimi-Movaghar V. (2012). Spinal tuberculosis: diagnosis and management. *Asian Spine Journal*.

[10] Kang M., Gupta S., Khandelwal N., Shankar S., Gulati M., Suri S. (1999). CT-guided fine-needle aspiration biopsy of spinal lesions. *Acta Radiologica*.

[11] Al-Khudairi N., Meir A. (2014). Isolated tuberculosis of the posterior spinal elements: case report and discussion of management. *JRSM Open*.

[12] Tuli S. M. (1975). Results of treatment of spinal tuberculosis by 'middle-path' regime. *Journal of Bone and Joint Surgery—Series B*.

[13] Patil V. R., Joshi A. R., Joshi S. S., Patel D. (2014). Lumbosacral actinomycosis in an immunocompetent individual: an extremely rare case. *Journal of Craniovertebral Junction and Spine*.

[14] Badali H., de Hoog G. S., Curfs-Breuker I., Klaassen C. H. W., Meis J. F. (2010). Use of amplified fragment length polymorphism to identify 42 *Cladophialophora* strains related to cerebral phaeohyphomycosis with in vitro antifungal susceptibility. *Journal of Clinical Microbiology*.

[15] Lyons M. K., Blair J. E., Leslie K. O. (2005). Successful treatment with voriconazole of fungal cerebral abscess due to *Cladophialophora bantiana*. *Clinical Neurology and Neurosurgery*.

[16] Dave B. R., Nanda A., Anandjiwala J. V. (2009). Transpedicular percutaneous biopsy of vertebral body lesions: a series of 71 cases. *Spinal Cord*.

[17] Aggarwal R., Dwivedi S., Aggarwal M. (2014). Unfamiliar manifestations of anti-tubercular therapy. *Journal of Family Medicine and Primary Care*.

[18] Riedel M G. (2010). Contaminación ambiental por Clostridium difficile. *Revista chilena de infectología*.

